# Down-Regulation of Tim-3 in Monocytes and Macrophages in *Plasmodium* Infection and Its Association with Parasite Clearance

**DOI:** 10.3389/fmicb.2017.01431

**Published:** 2017-08-02

**Authors:** Nan Hou, Ning Jiang, Yang Zou, Xianyu Piao, Shuai Liu, Shanshan Li, Qijun Chen

**Affiliations:** ^1^MOH Key Laboratory of Systems Biology of Pathogens, Institute of Pathogen Biology, Chinese Academy of Medical Sciences and Peking Union Medical College Beijing, China; ^2^Key Laboratory of Zoonosis, Shenyang Agriculture University Shenyang, China; ^3^Beijing Key Laboratory for Research on Prevention and Treatment of Tropical Diseases, Beijing Tropical Medicine Research Institute, Beijing Friendship Hospital, Capital Medical University Beijing, China

**Keywords:** malaria, Tim-3, monocytes, macrophages, *Plasmodium*

## Abstract

T-cell immunoglobulin and mucin-domain-containing molecule 3 (Tim-3) has complicated roles in regulating monocytes and macrophages in various diseases and it tends to be an inhibitory molecule to facilitate the immune escape of parasites in malaria. However, the mechanisms of Tim-3 mediated responses in monocytes and macrophages in malaria have not been clear. In this study, we found that *Plasmodium* infection down-regulated Tim-3 expression in peripheral monocytes of patients suffering from *Plasmodium falciparum* malaria and in splenic macrophages of *Plasmodium berghei* ANKA-infected mice. Tim-3 signal blockade with anti-Tim-3 antibodies enhanced phagocytosis and parasitical mediator production of murine splenic macrophages during *Plasmodium* infection. In conclusion, Tim-3 constricts monocytes/macrophages activity, and anti-Tim-3 treatment facilitates parasite clearance, especially in the early stage of *Plasmodium* infection.

## Introduction

Malaria is the most severe parasitic disease in the world, causing more than 200 million clinical cases and 438,000 deaths in 2015 (World Health Organization, 2015). *Plasmodium falciparum* (*P. falciparum*) causes the most lethal form of malaria and is responsible for ~95% of its mortality. Malaria infection induces strong innate immune responses in the host, which are necessary to initiate protective acquired immunity (Langhorne et al., 2004) and to result in direct antiparasitic effects (Sponaas et al., 2009). Monocytes and macrophages play important roles in parasite clearance, especially in immunologically naïve individuals lacking malaria-specific antibodies (Chua et al., 2013), though they also participate in the pathological events (Perkins et al., 2011; Chua et al., 2013). The precise mechanisms of these activities are still not clear, thus, the priorities of future research are clarifying the roles of these cells in malaria.

When exposed to a high number of parasites, circulating monocytes, and splenic macrophages have a central role in sensing and phagocytizing altered red blood cells. Opsonic and non-opsonic phagocytosis of parasite-infected erythrocytes by monocytes/macrophages is an effective way to reduce parasitaemia, while monocytes can initiate antibody-dependent cell inhibition to limit parasite growth (Chua et al., 2013). Monocytes/macrophages are also one of the main sources of parasiticidal mediators, including chemokine, and cytokines in malaria-infected individuals (Greve et al., 1999; Giribaldi et al., 2010). However, macrophages and monocytes have been shown to be the main contributors to the cytokine storm that is observed during acute malaria episodes (Giribaldi et al., 2010). Molecular interactions among monocytes, macrophages, and malaria parasites may alter the balance between protection and pathology in malaria-infected individuals (Perkins et al., 2011; Chua et al., 2013). Knowledge of the factors influencing the balance between protection and pathology can assist in the design of therapeutics aimed at modulating monocyte and macrophage function to improve outcomes.

T-cell immunoglobulin- and mucin-domain-containing molecule 3 (Tim-3) has been shown to be an important immunomodulatory molecule during the past decade. Although, Tim-3 was initially identified as a membrane marker specific for Th1 and Tc1 lymphocytes (Monney et al., 2002), its expression was soon confirmed in other immune cells, such as natural killer (NK) cells, dendritic cells, monocytes, and macrophages (Sakuishi et al., 2011). Tim-3 binding to its ligand galectin-9 has been proven to act as a negative regulatory pathway in T-cell (Sabatos et al., 2003; Sakai et al., 2010; Bi et al., 2011) and NK-cell (Ju et al., 2010; Hou et al., 2012) activation in many diseases. Studies have confirmed that monocytes from human peripheral blood in a quiescent state have high expression of Tim-3 with low cytokine production (Zhang et al., 2012; Ma et al., 2013), but the role of Tim-3 in monocytes/macrophages is complicated, as it varies in different diseases. It was reported that Tim-3 is able to promote macrophage activation in murine experimental and autoimmune encephalomyelitis (Anderson et al., 2007). Additionally, the Tim-3-galectin 9 interaction leads to macrophage activation and stimulates bactericidal activity (Jayaraman et al., 2010). However, Tim-3 was found to inhibit macrophage activation in murine acute Coxsackievirus B3-induced myocarditis (Frisancho-Kiss et al., 2006) and murine *Schistosoma japonicum* (*S. japonicum*) infection (Hou et al., 2015). Functional inhibition by Tim-3 was also detected in human peripheral blood CD14^+^ monocytes (Zhang et al., 2012).

*P. falciparum* and *Plasmodium vivax* infection causes increased Tim-3 expression in lymphocytes in the patients, leading to lymphocyte exhaustion (Costa et al., 2015; Hou et al., 2016), which reveals the role of Tim-3 in down-regulation of anti-malaria immunity. In our previous work, blocking Tim-3 signaling enhanced sterile immunity in *Plasmodium Berghei* ANKA (*Pb*ANKA)-infected C57BL/6 mice (Hou et al., 2016). However, it is not known whether Tim-3^+^ monocytes/macrophages are involved in this process. In the present study, we examined Tim-3 expression in monocytes in the peripheral blood of *P. falciparum*-infected patients as well as in splenic macrophages of *Pb*ANKA-infected mice, and the mechanism of Tim-3 regulation on monocytes/macrophages in the interaction between monocytes/macrophages and erythrocytic-stage parasites was further explored.

## Materials and methods

### Ethics statement

Human peripheral blood samples were donated by healthy volunteers and patients. The information of all individuals involved was anonymized. Written consent was obtained from all individuals for the publication of this study. All procedures performed on human samples were carried out in accordance with the tenets of the World Medical Association's Declaration of Helsinki. All procedures performed on the animals in this study were conducted according to the animal husbandry guidelines of the Chinese Academy of Medical Sciences. Studies on humans and animals were reviewed and approved by the Ethical Committee and the Experimental Animal Committee of the Chinese Academy of Medical Sciences.

### Patients

Twenty-one patients suffering from falciparum malaria (FM) and 16 healthy individuals were recruited at Beijing Friendship Hospital at Capital Medical University from March 2015 to August 2016. Peripheral blood and plasma were obtained from all the subjects. The characteristics of the patients and healthy individuals are summarized in Supplementary Table 1. All FM patients had primary infections, documented by Giemsa-stained thin blood smears for parasite identification and confirmed by nest PCR that targets variant sequences in the small subunit ribosomal RNA genes (Kimura et al., 1997). All samples were obtained before treatment.

### *Pb*ANKA infection and anti-Tim-3 treatment

Six-week-old male C57BL/6 mice (special pathogen free) were purchased from Vital River Laboratory Animal Technology Co., Ltd. (Beijing, China). All mice were maintained in a pathogen-free facility and randomly divided into groups. *Pb*ANKA-infected mice were constructed as previously described (Hou et al., 2016), and they were infected intraperitoneally with 10^6^ parasitized red blood cells. For anti-Tim-3 treatment, the mice received one intraperitoneal injection of 100 μg of anti-Fc antibody (eBioscience, San Diego, USA) to reduce non-specific binding, followed by treatment with either 100 μg of purified anti-mouse Tim-3 antibody (catalog no. 14-5870, eBioscience) or purified rat IgG2α K isotype control (catalog no. 14-4321-85, eBioscience) once a day after infection. Parasitaemia was also monitored daily by examining tail blood smears stained with Giemsa stain (Sigma-Aldrich, St. Louis, MO, USA). The smears were observed using a digital camera and analyzed using the Image-Pro Plus 6.0 software.

### Cell preparation and isolation

Murine spleens were cut into pieces, minced, and pressed through 200-gauge stainless steel mesh. Then, the red blood cells (RBCs) were depleted with a red blood cell lysis solution as previously described (Hou et al., 2016). Murine splenic mononuclear cells were isolated by gradient centrifugation with HISTOPAQUE-1083 (Sigma-Aldrich) according to the manufacturer's instruction. Macrophages were isolated from murine splenic mononuclear cells using CD11b Microbeads (Miltenyi, Bergisch-Gladbach, Germany) according to the manufacturer's protocol. This procedure consistently yields a population of purified macrophages (>90% CD11b^+^ by flow cytometry) with more than 98% viability, as indicated by trypan blue exclusion. The mouse RBCs were enriched by gradient centrifugation using HISTOPAQUE-1083.

### Flow cytometry

Flow cytometry was conducted as previously described (Hou et al., 2016). The antibodies used in this study were as follows: Anti-Human CD14 PerCP-Cy5.5, mouse IgG1κ isotype control PerCP-Cy5.5, anti-mouse CD11b FITC, rat IgG2a K isotype control FITC, anti-mouse TIM-3 PE, and rat IgG2α K isotype control PE (all obtained from eBioscience, San Diego, CA, USA), anti-human Tim-3-PE, and rat IgG_2A_ isotope control-PE (both obtained from R&D Systems). The cells were detected and analyzed using a FACS Canto II flow cytometer (BD Biosciences, San Jose, CA, USA), and the gates for positive cells were defined using the isotype controls.

### *In vitro* anti-Tim-3 treatment assays

Murine splenic mononuclear cells or splenic CD11b^+^ cells were cultured in RPMI 1640 medium (HyClone, Thermo, Beijing, China) supplemented with 10% fetal bovine serum (GIBCO, Grand Island, USA) and plated at 5 × 10^5^ cells per well in 96-well polystyrene plates. Three micrograms or milliliters of the anti-mouse TIM-3 purified antibody were simultaneously added and incubated for 30 min to block the Tim-3 signal pathway. Purified rat IgG2α K isotype was used as a control antibody. *Pb*ANKA-infected red blood cells with a parasitaemia >30% (*Pb*-iRBC) were then added into the indicated wells at a concentration of 1 × 10^6^ per well.

Phagocytosis was assessed by incubating murine mononuclear cells with or without Tim-3 antibodies in suspension for 4 h with *Pb*-iRBC prestained using the CellTrace™ Far Red Cell Proliferation Kit (Thermo Fisher Scientific, Carlsbad, CA, USA), according to the manufacturer's instruction. The frequencies of total monocytes/macrophages positive for Far Red were determined by flow cytometry.

Murine splenic CD11b^+^ cells treated with or without anti-Tim-3 antibodies were co-cultured with *Pb*-iRBC or RBCs from uninfected mice (uRBC) to detect parasiticidal mediator production. The cells were incubated for 24 h and then collected for subsequent experiments. The supernatants were harvested and maintained at −80°C. The levels of tumor necrosis factor (TNF-α) and nitric oxide (NO) in human plasma, mouse serum and cultured cell supernatants from murine CD11b^+^ cells were determined using the Human TNF-α ELISA Kit, the Mouse TNF-α ELISA Kit (all from R&D Systems) and the Total Nitric Oxide Assay Kit (Beyotime Biotechnology, Shanghai, China), respectively, according to the manufacturer's instructions.

### Real-time quantitative reverse-transcription PCR (QRT-PCR)

Quantitative real-time PCR was performed to analyse the expression of genes in both human and mouse immune cells. The details of the QRT-PCR assay were previously described (Hou et al., 2015). The primers for the genes were derived from the Primer Bank (https://pga.mgh.harvard.edu/primerbank/) and are listed in Supplementary Table 2. The relative expression level of each gene was analyzed using SDS 1.4 software (Applied Biosystems, Carlsbad, USA).

### Western blot

CD11b^+^ cells were lysed with lysis buffer (Cytobuster, Novagen, San Diego, CA) for 30 min on ice, The mixture was centrifuged at 12,000 rpm for 30 min at 4°C and protein concentrations were quantified by the BCA protein Assay Kit (Pierce, Rockford, IL, USA). Fifty micrograms of protein of each sample was separated by SDS-PAGE and transferred onto polyvinylidene difluoride membranes (Millipore, Bedford, MA, USA). After blocking with 5% dried milk (wt/vol) in PBS for 1 h at room temperature, each membrane was incubated in the same buffer with CD36 monoclonal Antibody, CD54 (ICAM-1) Monoclonal Antibody (all from ThermoFisher Scientific, Rockford, IL, USA, diluted 1:1,000) or IgG control for overnight at 4°C, this was followed by washing and subsequent incubation with IRDye 800 CW Conjugated goat Anti-mouse IgG (H+L) antibody (Li-COR Biosciences, Lincoln, Nebraska, USA), and detection were carried out using Odyssey (Li-COR).

### Statistical analysis

The data were analyzed using the GraphPad Prism 5.0 software and Microsoft Excel 2007. The results were analyzed using a 2-tailed paired *t*-test. The Wilcoxon test for paired samples was used for the data that did not fit a Gaussian distribution. Values of *p* < 0.05 were considered significant.

## Results

### *Plasmodium* infection leads to increased quantity and decreased Tim-3 expression in monocytes/macrophages

**Figure 1:**
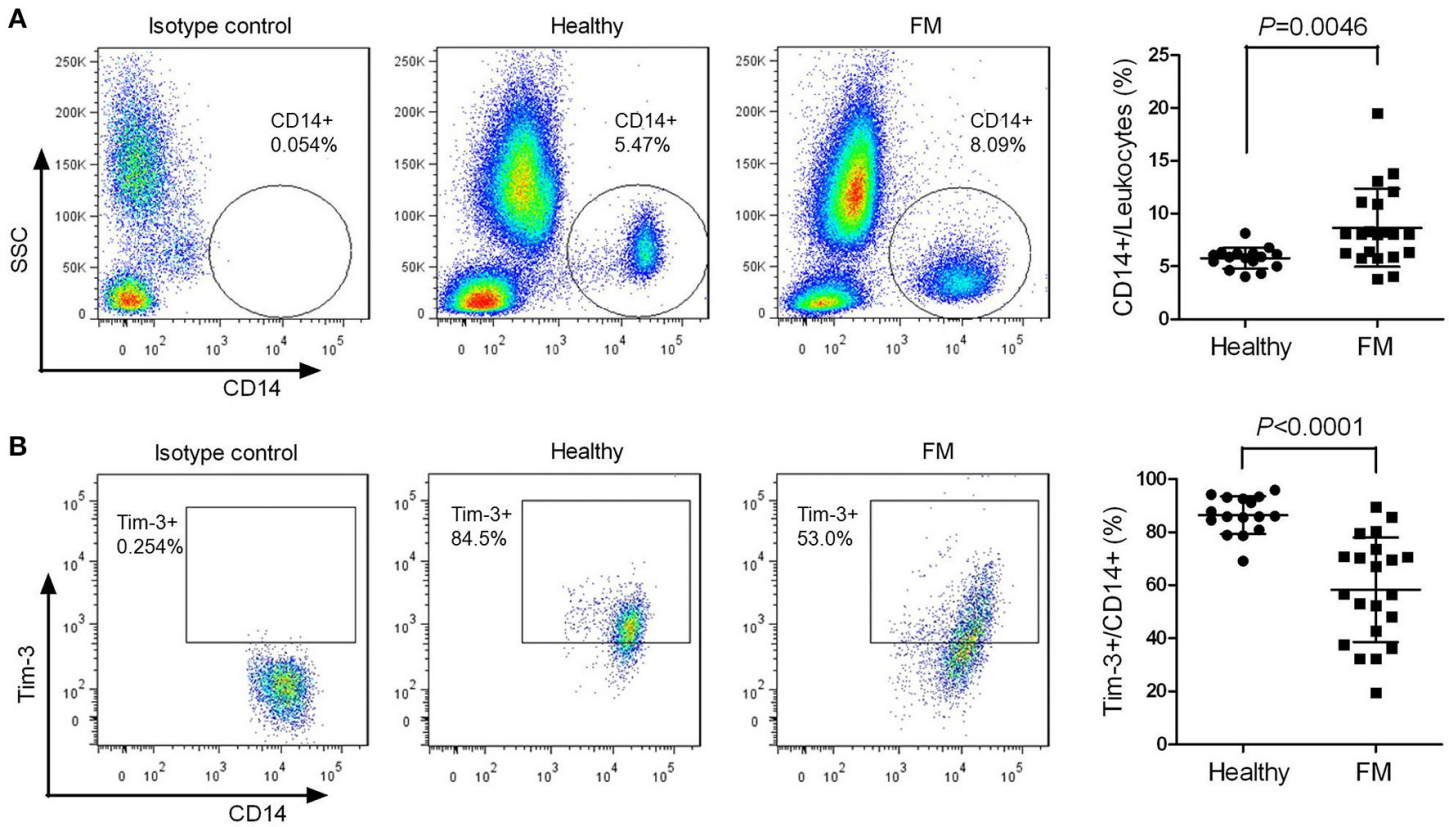
Decreased T-cell immunoglobulin- and mucin-domain-containing molecule 3 (Tim-3) expression in monocytes from falciparum malaria (FM) patients. Peripheral blood from 21 patients with FM and 16 healthy individuals (Healthy) were analyzed by flow cytometry. **(A)** Representative dot plots (left panels) and a scatter plot (right panel) showing the proportion of CD14^+^ cells relative to the total leukocyte populations. **(B)** Representative dot plots (left panels) and a scatter plot (right panel) showing the frequency of Tim-3-expressing cells in the CD14^+^ cell populations. Each dot in the scatter plot represents one individual, with horizontal lines indicating the mean ± *SD*.

The quantity of peripheral monocytes and the expression of Tim-3 in monocytes of patients suffering from FM and healthy individuals were examined by flow cytometry. The proportion of CD14^+^ cells in peripheral leukocytes of FM patients was much higher than that of healthy individuals (Figure 1A), and the frequency of Tim-3^+^ cells in CD14^+^ cells was significantly decreased in FM patients (Figure 1B).

**Figure 2:**
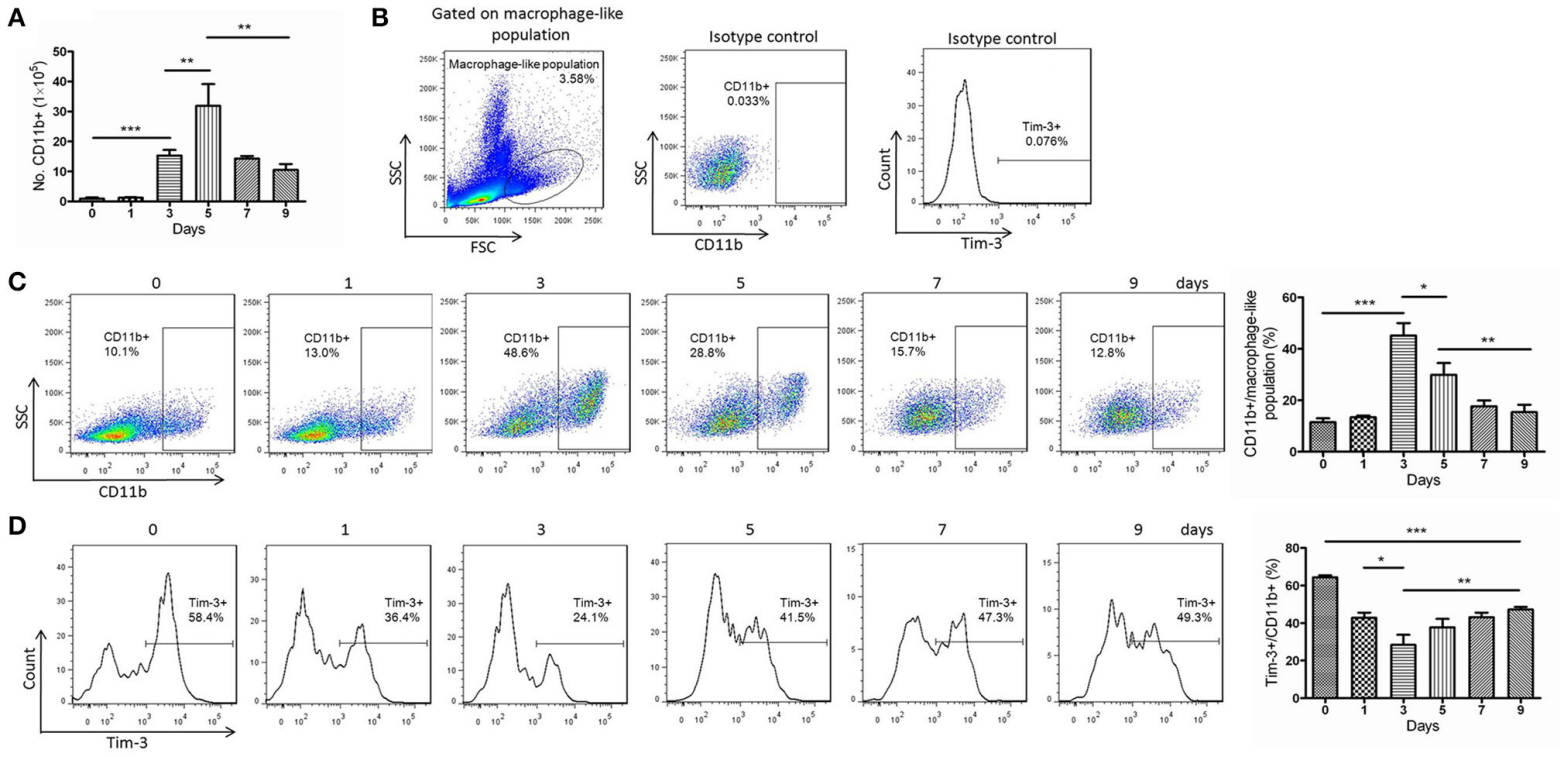
Decreased T-cell immunoglobulin- and mucin-domain-containing molecule 3 (Tim-3) expression in splenic CD11b^+^ cells from *Plasmodium berghei* ANKA (*Pb*ANKA)-infected mice. Splenic cells of *Pb*ANKA-infected C57BL/6 mice were collected at days 0, 1, 3, 5, 7, and 9 post-infection. **(A)** CD11b^+^ cells were isolated from splenic mononuclear cells and manually counted. **(B**–**D)** The CD11b^+^ populations and the Tim-3 expression were measured using flow cytometry. **(B)** The gate strategies and isotype controls. **(C)** Representative dot plots (left panel) and a histogram (right panel) showing the frequency of CD11b^+^ cells in the macrophage-like population. **(D)** Representative dot plots (left panel) and a histogram (right panel) showing the frequency of Tim-3-expressing cells in the CD11b^+^ macrophage populations. The results are representative of 3 independent experiments with five to seven mice in each group per experiment, with data indicating the mean + *SD*. ^*^p < 0.05, ^**^p < 0.01, and ^***^p < 0.0001.

*Pb*ANKA is lethal in mice and has been widely used as experimental models for studies of human malaria diseases (Troye-Blomberg et al., 1994). Thus, we used *Pb*ANKA to study macrophages in murine malaria. The number of CD11b^+^ macrophages in the spleens of *Pb*ANKA-infected mice, counted manually, peaked at day 5 post-infection (Figure 2A). The proportion of splenic CD11b^+^ macrophages in the macrophage-like population reached a peak at day 3 post-infection (Figure 2C). However, the quantity of CD11b^+^ macrophages did not continuously increase. After the peak, both the number and the proportion started to decrease, but the number of splenic CD11b^+^ macrophages at day 9 was still much higher than that at day 0 (day 0, 0.94 ± 0.36% vs. day 9, 10.53 ± 1.94%, *p* = 0.0011, Figure 2A). Tim-3 expression in splenic CD11b^+^ macrophages of *Pb*ANKA-infected mice sharply decreased until day 3 post-infection and then gradually increased (Figure 2D). The Tim-3 expression in CD11b^+^ macrophages at day 9 post-infection was still lower than that at day 0 (day 0, 64.37 ± 1.05% vs. day 9, 47.27 ± 1.39%, *p* < 0.0001, Figure 2D).

### Tim-3 signal blockade improved the phagocytosis of macrophages

**Figure 3:**
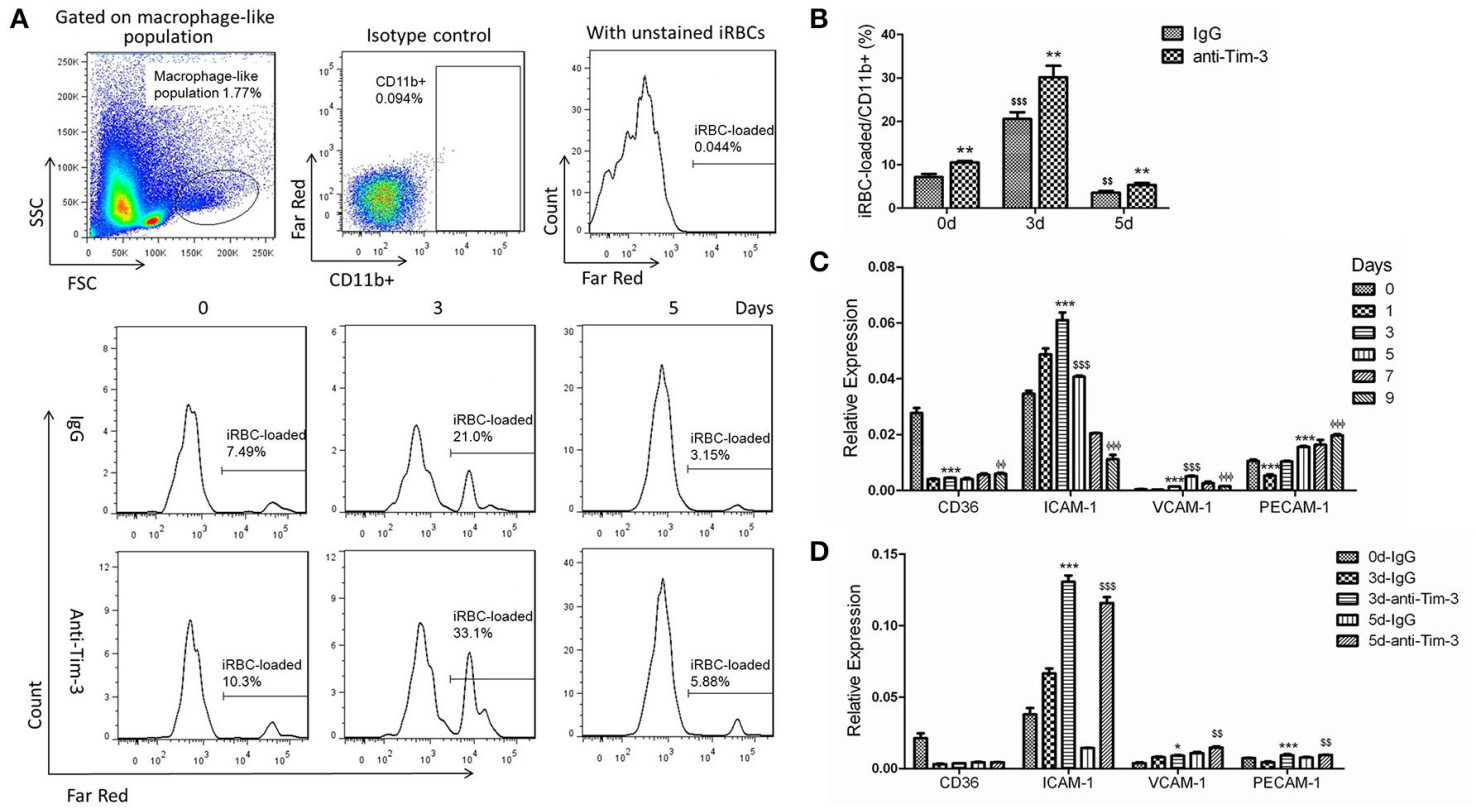
T-cell immunoglobulin- and mucin-domain-containing molecule 3 (Tim-3) signal blockade elevated the phagocytic ability of splenic macrophages during *Plasmodium berghei* ANKA (*Pb*ANKA) infection. **(A,B)** *Pb*ANKA-infected murine red blood cells (iRBCs) with a parasitaemia >30% were CellTrace™ Far Red-labeled. Splenic mononuclear cells from *Pb*ANKA-infected mice at days 0, 3, and 5 post-infection (0, 3, and 5 d) were treated with anti-Tim-3 antibodies (anti-Tim-3) or IgG control (IgG), and co-cultured with stained or unstained iRBCs. The mean fluorescence intensity of Far Red within CD11b^+^ cells from the macrophage-like population was measured by flow cytometry after 4 h of cultivation. **(A,B)** Representative histograms **(A)** and comparisons among groups **(B)** of the frequency of Far Red-stained iRBC-loaded cells in CD11b^+^ macrophage populations. **(C,D)** CD11b^+^ macrophages were isolated from splenic mononuclear cells of *Pb*ANKA-infected mice at days 0, 1, 3, 5, 7, and 9 post-infection **(C)**, or they were isolated from anti-mouse Tim-3 antibody- or IgG control-treated *Pb*ANKA-infected mice at days 0, 3, and 5 post-infection **(D)**. The expression of adhesion molecules, including cluster of differentiation 36 (CD36), intercellular adhesion molecule (ICAM)-1, vascular cell adhesion molecule (VCAM)-1, and platelet endothelial cell adhesion molecule (PECAM)-1, in these cells was detected by real-time RT-PCR. Gene expression was normalized against β-tubulin and is presented as the fold-change vs. the expression of GAPDH from the cells at day 0. **(B)** ^*,$^Indicate comparisons to the IgG group or IgG group of day 0, respectively, **(C)** ^*,$,Φ^Indicate compared to day 0, day 3, or day 5, respectively. **(D)** ^*,$^Indicate compared to 3 d-IgG or 5 d-IgG, respectively. The results are representative of two independent experiments with five to seven mice in each group with data indicating the mean + *SD*, ^*,$,Φ^*p* < 0.05, ^**,$$,ΦΦ^*p* < 0.01, ^***,$$$,ΦΦΦ^*p* < 0.0001.

*Pb*-iRBCs previously stained with CellTrace™ Far Red was used to quantify the level of phagocytosis of macrophages from *Pb*ANKA-infected mice. Splenic CD11b^+^ macrophages from mice at day 3 post-infection displayed significantly higher levels of phagocytosis of *Pb*-iRBCs compared to those from mice at day 0. However, the phagocytic ability of CD11b^+^ macrophages at day 5 post-infection sharply declined and was even lower than those at day 0 (Figures 3A,B). Tim-3 signal blockade with the anti-Tim-3 antibody effectively elevated the phagocytosis of *Pb*-iRBCs by macrophages, especially at day 3 post-infection (Figures 3A,B). Adhesion molecules, including cluster of differentiation 36 (CD36), intercellular adhesion molecule (ICAM)-1, vascular cell adhesion molecule (VCAM)-1 and platelet endothelial cell adhesion molecule (PECAM)-1, may favor the binding and uptake of parasite-infected RBCs by macrophages (Carvalho et al., 2010; Antonelli et al., 2014). The expression of the coding genes of splenic macrophages during the infectious periods was detected by QRT-PCR. The four molecules displayed different expression patterns. The level of CD36 sharply decreased as soon as *Pb*ANKA infection initiated. The levels of ICAM-1 and VCAM-1 increased to a peak and then gradually decreased. The level of PECAM-1 declined at day 1 post-infection and then gradually increased. The expression of ICAM-1 was much higher than that of the other genes during the first 5 days post-infection (*p* < 0.0001, Figure 3C). Similar result of CD36 expression in *Pb*ANKA-infected splenic CD11b^+^ macrophages were obtained by Western Blot, while the protein level of ICAM-1 decreased as soon as *Pb*ANKA infection initiated and then gradually increased to a peak at day 5 (Supplementary Figure 1A). To investigate the relationship of Tim-3 and these adhesion molecules, Tim-3 antibodies were applied to block the Tim-3 signaling pathway in mice models. *Pb*ANKA-infected mice received one intraperitoneal injection of 100 μg of an anti-mouse Tim-3 antibody or the IgG control every other day following infection. The splenic CD11b^+^ macrophages were obtained at days 0, 3, and 5 post-infection. The QRT-PCR results showed that the splenic CD11b^+^ macrophages from anti-Tim-3 antibody treated mice had higher expression levels of ICAM-1, VCAM-1, and PECAM-1 compared to the control groups (Figure 3D). Similar results of CD36 and ICAM-1 expression in splenic CD11b^+^ macrophages from *Pb*ANKA-infected mice were obtained by Western Blot (Supplementary Figure 1B). Thus, ICAM-1 was the most susceptible gene, while CD36 was not influenced by Tim-3 antibodies.

### Tim-3 signal blockade elevated the production of parasiticidal mediators

**Figure 4:**
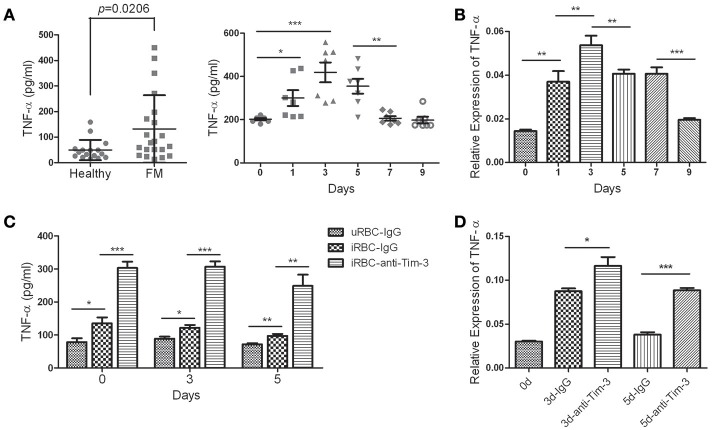
T-cell immunoglobulin- and mucin-domain-containing molecule 3 (Tim-3) signal blockade promoted tumor necrosis factor (TNF)-α production by macrophages during malaria infection. **(A)** The TNF-α concentration in the plasma from 21 patients with falciparum malaria (FM) and 16 healthy individuals (Healthy, left panel) and in the sera from *Pb*ANKA-infected mice at days 0, 1, 3, 5, 7, and 9 post-infection (right panel) were detected using enzyme-linked immunosorbent assays (ELISAs). Each dot in the scatter plot represents one individual or one mouse, with horizontal lines indicating the mean ± *SD*. **(B**,**C)** Splenic CD11b^+^ macrophages were obtained from *Pb*ANKA-infected mice at days 0, 1, 3, 5, 7, and 9 post-infection. **(B)** The TNF-α expression in these cells was detected by real-time RT-PCR. **(C)** CD11b^+^ macrophages at days 0, 3, and 5 post-infection were pretreated with anti-Tim-3 antibodies (anti-Tim-3) or IgG control (IgG), and then, the cells were co-cultured with *Pb*ANKA-infected red blood cells (iRBC) for 24 h or uninfected red blood cells (uRBC) as a control. The TNF-α level in the supernatant was detected by ELISA. **(D)** *Pb*ANKA-infected mice were treated with the anti-mouse Tim-3 antibody (anti-Tim-3) or IgG control (IgG). Splenic CD11b^+^ macrophages were obtained at days 0, 3 and 5 post-infection (0, 3, and 5 d), and the TNF-α expression in these cells were detected by real-time RT-PCR. **(B,D)** The gene expression was normalized against β-tubulin and is presented as the fold-change vs. the expression of GAPDH of cells from day 0. The right panel of **(A–D)**. The results are representative of two independent experiments with 5–7 mice in each group. **(B–D)** The data indicates the mean + *SD*, ^*^p < 0.05, ^**^p < 0.01, ^***^p < 0.0001.

TNF-α is the key effector molecule released mainly by activated monocyte/macrophages during malaria (Giribaldi et al., 2010). The ELISA results showed that the level of TNF-α in the plasma of FM patients was much higher than that of healthy individuals (Figure 4A left panel) and the level of TNF-α in the sera of *Pb*ANKA-infected mice, detected along the infectious periods, quickly elevated to a peak at day 3 post-infection and then started to decline (Figure 4A right panel). The splenic CD11b^+^ macrophages from mice 0, 1, 3, 5, 7, and 9 days post-infection were obtained to detect the mRNA expression of TNF-α. The QRT-PCR results showed that variation in TNF-α mRNA expression in splenic CD11b^+^ macrophages was consistent with the variation in the TNF-α concentration in the sera of infected mice (Figure 4B). Our *in vitro* assay showed that splenic CD11b^+^ cells from *Pb*ANKA-infected mice at days 0, 3, and 5 post-infection co-cultured with *Pb*-iRBC had higher TNF-α production in the supernatant compared to that co-cultured with uRBC, and anti-Tim-3 antibody treatment further promoted TNF-α production (Figure 4C). *In vivo* assays were also carried out, and splenic CD11b^+^ cells from the anti-mouse Tim-3 antibody- or IgG control-treated *Pb*ANKA mice were obtained at days 0, 3, and 5 post-infection. The QRT-PCR results showed that the anti-Tim-3 treated groups had higher expression levels of TNF-α compared to that of the control groups (Figure 4D).

**Figure 5:**
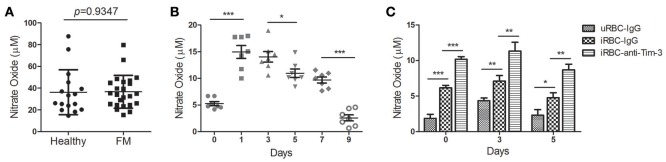
T-cell immunoglobulin- and mucin-domain-containing molecule 3 (Tim-3) signal blockade promoted nitric oxide (NO) production of macrophages during malaria. **(A**,**B)** The levels of NO in the plasma from 21 patients with falciparum malaria (FM) and 16 healthy individuals (Healthy, **A**) and in the serum from *Pb*ANKA-infected mice at days 0, 1, 3, 5, 7, and 9 post-infection **(B)** were detected. Each dot in the scatter plot represents one individual or one mouse, with horizontal lines indicating the mean ± *SD*. **(C)** Splenic CD11b^+^ macrophages from *Pb*ANKA-infected mice at days 0, 3, and 5 post-infection were pretreated with anti-Tim-3 antibodies (anti-Tim-3) or IgG control (IgG), and then co-cultured with *Pb*ANKA-infected erythrocytes (iRBC) for 24 h. Uninfected red blood cells (uRBC) were used as a control. The level of NO in the supernatant was detected, with the data indicating the mean + *SD*. **(B,C)** The results are representative of two independent experiments with five to seven mice in each group, ^*^p < 0.05, ^**^p < 0.01, ^***^p < 0.0001.

NO is also a key effector molecule made by activated monocyte/macrophages (Greve et al., 1999). The level of NO in the plasma was not significantly different between FM patients and healthy individuals (Figure 5A). The NO concentration in the plasma from *Pb*ANKA-infected mice sharply increased to a peak at day 1 post-infection and then quickly decline (Figure 5B). Serum samples were obtained from *Pb*ANKA-infected mice treated with anti-Tim-3 antibodies or IgG control, and the NO level was not different between the two groups (data not shown). Our *in vitro* assay showed that splenic CD11b^+^ cells from *Pb*ANKA-infected mice co-cultured with *Pb*-iRBC had higher NO production in the supernatant compared to those co-cultured with uninfected RBC, and anti-Tim-3 treatment further promoted the NO production (Figure 5C).

### Tim-3 induces alternative macrophage activation during malaria

**Figure 6:**
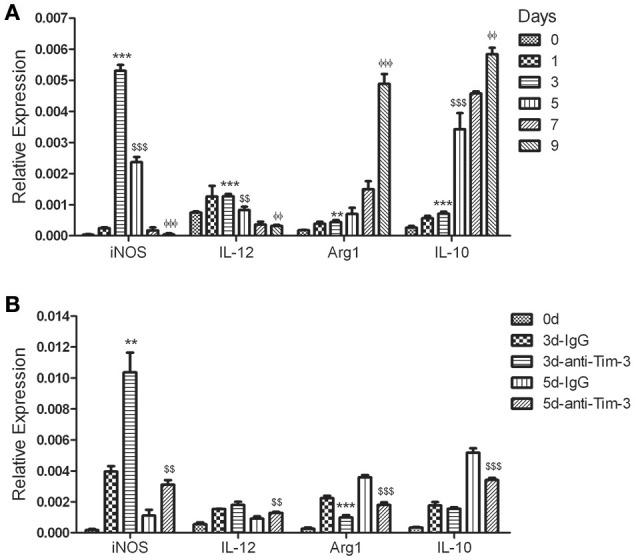
T-cell immunoglobulin- and mucin-domain-containing molecule 3 (Tim-3) signal blockade polarized macrophages from the M2 phenotype to the M1 phenotype during *Plasmodium berghei* ANKA (*Pb*ANKA) infection. CD11b^+^ macrophages were isolated from splenic mononuclear cells of *Pb*ANKA-infected mice at days 0, 1, 3, 5, 7, and 9 post-infection **(A)** or anti-mouse Tim-3 antibody- or IgG control-treated *Pb*ANKA-infected mice at days 0, 3, and 5 post-infection (d). The expression of the key markers of classically activated macrophages, inducible nitric oxide synthase (iNOS) and IL (interleukin) −12, and alternatively-activated macrophages, arginase-1 (Arg1) and IL-10, in these cells was detected by real-time RT-PCR. Gene expression was normalized against β-tubulin and is presented as the fold-change vs. the expression of GAPDH in the cells from day 0. **(A)** ^*,$,Φ^Indicate compared to days 0, 3, or 5, respectively. **(B)** ^*,$^Indicate compared to 3 d-IgG or 5 d-IgG, respectively. The results are representative of two independent experiments with 5–7 mice in each group, with horizontal lines indicating the mean ± *SD*. ^**,$$,ΦΦ^*p* < 0.01, ^***,$$$,ΦΦΦ^*p* < 0.0001.

The splenic CD11b^+^ macrophages from mice at 0, 1, 3, 5, 7, and 9 days post-infection were obtained and used to detect the key markers of classically activated macrophages, inducible nitric oxide synthase (iNOS) and IL (interleukin)-12, and the key markers of alternatively activated macrophages, arginase-1 (Arg1) and IL-10 (Murray et al., 2014). The QRT-PCR results showed that the expression of iNOS and IL-12 peaked at day 3 post-infection and then declined, while the expression of Arg1 and IL-10 slowly increased in the first 3 days and rapidly increased in the following days (Figure 6A). To investigate the effect of Tim-3 on macrophage polarization, splenic CD11b^+^ cells from *Pb*ANKA-infected mice treated with the anti-Tim-3 antibodies or IgG control were obtained at days 0, 3, and 5 post-infection. Real-time PCR exhibited that the Tim-3 signal blockade by anti-Tim-3 antibodies promoted the expression of iNOS and IL-12 and inhibited the expression of Arg1 and IL-10 (Figure 6B).

## Discussion

Elevated expression of Tim-3 has been shown to induce lymphocyte exhaustion, and anti-Tim-3 treatment inhibited the splenomegaly induced by *Pb*ANKA infection, while elevated the activity of lymphocytes, thus resulted in accelerated clearance of the parasites, reduced neurological signs associated with experimental cerebral malaria and prolonged survival time of *Pb*ANKA-infected mice (Hou et al., 2016). These results indicated the pivotal role of well-balanced immune cell ability in parasite clearance in *Plasmodium* infection. However, the role of Tim-3 in regulation of monocytes/macrophages responses in malaria has not been studied, even though circulating monocytes, and splenic macrophages have a central role in parasite clearance, as they are exposed to a high number of parasites (Chua et al., 2013). Thus, in the present study, we investigated the role of Tim-3 in regulating monocytes/macrophages from human peripheral blood and mouse spleens during malaria, especially in the early stage of infection.

Monocytes/macrophages play important roles in parasite clearance, especially in immunologically naïve individuals lacking malaria-specific antibodies (Chua et al., 2013). We observed significant increase of monocytes/macrophages in the early stage of *Plasmodium* infection. At the same time, Tim-3 expression was sharply decreased to liberate monocytes/macrophages for activation (Figures 1, 2), and the macrophages possessed the powerful abilities of phagocytosis and anti-parasite mediator production (Figures 3–6). The amount and activity of lymphocytes tends to decrease during *Plasmodium* infection (Hou et al., 2016); thus, their increased quantity and activity indicates the importance of monocytes and macrophages in anti-malaria immunity, especially in the early clearance of parasites.

The role of Tim-3 in monocytes/macrophages is complicated, as it displayed various regulatory functions in different diseases, and this study revealed an inhibitory role of Tim-3 in monocytes/macrophages in malaria. We found both human peripheral monocytes and murine splenic macrophages have a high expression of Tim-3 in the quiescent state (Figures 1, 2), which is consistent with previous reports (Zhang et al., 2012; Ma et al., 2013). *Plasmodium* infection could reduce Tim-3 expression in monocytes/macrophages in the early stage of the disease (Figures 1, 2) with an increased activity of these cells (Figures 3–6). However, the rapidly increased parasitaemia soon reversed the expression of Tim-3 to a relatively higher extent and induced the functional inhibition of macrophages. Tim-3 signal blockade with anti-Tim-3 antibodies effectively enhanced phagocytosis (Figure 3) and promoted TNF-α and NO production in murine splenic macrophages both *in vivo* and *in vitro* (Figures 4, 5), without elevating the serum TNF-α (Hou et al., 2016) and NO levels (data not shown) in *Pb*ANKA-infected mice. These results, combined with our previous work (Hou et al., 2016), indicated that Tim-3 could inhibit the activity of both lymphocytes and macrophages and highlighted the value of anti-Tim-3 treatment in anti-malaria strategies.

Non-opsonic phagocytosis by direct binding of whole IEs to monocyte and macrophage phagocytic receptors is an important factor regulating the efficiency of monocytes/macrophages mediated phagocytosis (Chua et al., 2013). The scavenger receptor CD36 is directly involved in the non-opsonic uptake of IEs (Udomsangpetch et al., 1997; McGilvray et al., 2000; Ayi et al., 2005). However, our results found that the CD36 expression in splenic macrophages from *Pb*ANKA-infected mice was sharply decreased as soon as the first day after infection (Figure 3C). This presumably can prevent early clearance of IEs by monocytes/macrophages and increase susceptibility to malaria. In addition to CD36, various adhesion molecules, including ICAM-1, VCAM-1, and PECAM-1, have also been described as important receptors that bind *P. falciparum*-infected red blood cells and influence the outcome of disease (Newbold et al., 1997; Rowe et al., 2009). Although these adhesion molecules were originally identified as endothelium receptors for parasitized red blood cells, their expression on monocytes, and macrophages may favor the binding and uptake of parasite-infected RBCs (Carvalho et al., 2010; Antonelli et al., 2014). Our studies revealed the dynamic changes in these adhesion molecules, and the expression level of ICAM-1 was the highest, with a peak at day 3 post-infection by QRT-PCR and day 5 post-infection by Western Blot (Figure 3C, Supplementary Figure 1A). Thus, ICAM-1 is probably the key molecule in the uptake of IEs by macrophages during *Pb*ANKA infection. A recent study reported similar results in that the phagocytic activity of the monocytes positively correlated with the expression of ICAM-1, PECAM-1, and LFA-1 and the blockade of each of these adhesion molecules efficiently inhibited the phagocytosis of *P. vivax-*infected reticulocytes (Antonelli et al., 2014). Anti-Tim-3 treatment substantially enhanced the expression of ICAM-1 and slightly increased the expression levels of VCAM-1 and PECAM-1; however, it did not influence the expression of CD36 (Figure 3D, Supplementary Figure 1B), indicating the important role of ICAM-1, but not CD36, in Tim-3 signal blockade mediated elevation of phagocytosis in macrophages.

Tim-3 was reported to engage in alternative macrophage activation (Hou et al., 2015). Classically activated macrophages (M1 macrophages) participate in phagocytosis, secrete antimicrobial molecules, and activate adaptive immune responses; alternatively activated macrophages (M2 macrophages) participate in the resolution of inflammation after infection (Murray et al., 2014). Our results showed that the splenic macrophages of *Pb*ANKA-infected mice were M1 type dominant with a higher expression of inducible nitric oxide (iNOS) and interleukin-12 (IL-12) in the first 3–5 days post-infection and then tended to be M2 dominant with increased expression of arginase 1 (Arg1) and IL-10 (Figure 6). Thus, anti-Tim-3 treatment with anti-Tim-3 antibodies promoted classical macrophage activation and consequently induce active immunity against parasites.

In conclusion, *Plasmodium* infection generally down-regulated Tim-3 expression in monocytes/macrophages, a short phase decreased Tim-3 expression and increased number of monocytes/macrophages in the early stage of infection led to monocytes/macrophages activation and played important roles in early parasite clearance. Further, the blockade of Tim-3 signaling enhanced phagocytosis and parasiticidal mediator production of macrophages and prevented alternative macrophage activation. Thus, an anti-Tim-3 strategy may improve monocytes/macrophages-mediated immunity against *Plasmodium* and facilitate the development of new therapeutic interventions.

## Author contributions

NH and QC designed the study and wrote the manuscript. NH and NJ performed the main experiments. YZ collected the clinical samples and performed partial experiments. XP, SLiu, and SLi provided laboratory assistance to the study.

### Conflict of interest statement

The authors declare that the research was conducted in the absence of any commercial or financial relationships that could be construed as a potential conflict of interest.

## Supplementary material

Supplementary Figure 1 Anti-Tim-3 treatment upregulated ICAM-1 expression of splenic macrophages during *Plasmodium berghei* ANKA (*Pb*ANKA) infection. CD11b+ macrophages were isolated from splenic mononuclear cells of *Pb*ANKA-infected mice at days 0, 1, 3,5, 7 and 9 post-infection **(A)**, or they were isolated from anti-mouse Tim-3 antibody- (anti-Tim-3) or IgG control-treated *Pb*ANKA-infected mice at days 0, 3 and 5 post-infection **(B)**. The expression of adhesion molecules, including cluster of differentiation 36 (CD36) and intercellular adhesion molecule (ICAM)-1 in these cells were detected by Western Blot, β-actin as control.

Supplementary Table 1 Clinical characteristics of the enrolled subjects.

Supplementary Table 2 Primers for murine genes.
