# Supplementary figures and images for: Down-Regulation of Tim-3 in Monocytes and Macrophages in Plasmodium Infection and Its Association with Parasite Clearance
Source: Front Microbiol. 2017 Aug 2;8:1431. doi: 10.3389/fmicb.2017.01431 (PMC5539084; doi:10.3389/fmicb.2017.01431)

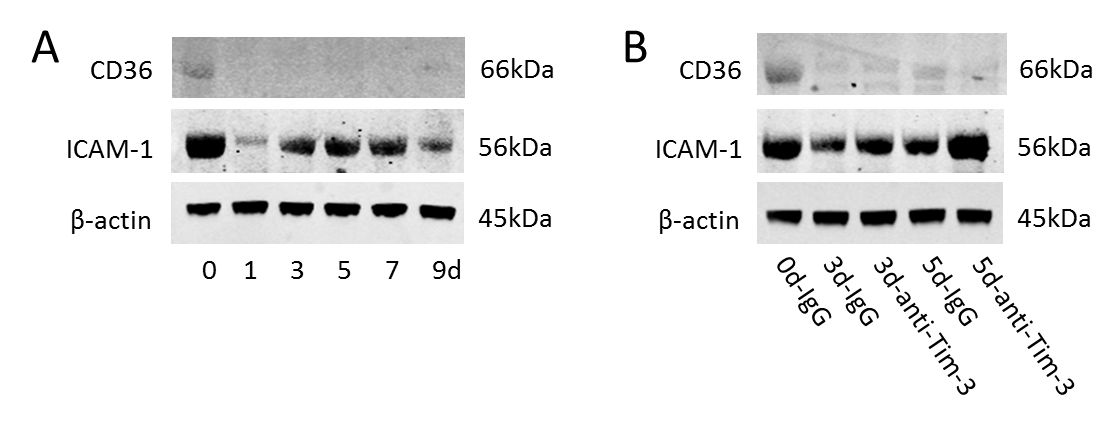

Supplement: Supplementary Figure 1 — Anti-Tim-3 treatment upregulated ICAM-1 expression of splenic macrophages during Plasmodium berghei ANKA (PbANKA) infection. CD11b+ macrophages were isolated from splenic mononuclear cells of PbANKA-infected mice at days 0, 1, 3,5, 7 and 9 post-infection (A), or they were isolated from anti-mouse Tim-3 antibody- (anti-Tim-3) or IgG control-treated PbANKA-infected mice at days 0, 3 and 5 post-infection (B). The expression of adhesion molecules, including cluster of differentiation 36 (CD36) and intercellular adhesion molecule (ICAM)-1 in these cells were detected by Western Blot, β-actin as control. [file Image1.JPEG]
