# Supplementary material for: Down-Regulation of Tim-3 in Monocytes and Macrophages in Plasmodium Infection and Its Association with Parasite Clearance
Source: Front Microbiol. 2017 Aug 2;8:1431. doi: 10.3389/fmicb.2017.01431 (PMC5539084; doi:10.3389/fmicb.2017.01431)
Supplement: Supplementary Table 1 — Clinical characteristics of the enrolled subjects. [file Table1.DOC]

Supplemental Table 1. Clinical characteristics of the enrolled subjects.

| Subjects | FM  (n = 21) | Health  (n = 16) |
| --- | --- | --- |
| Age mean^*^ (years)  Age range (years)  Male/Female  Race  Input Region | 35.84±10.93  23-53  21/0  Chinese  Angola 3, Burundi 1, Ghana 1, Myanmar 3, Mozambique 1, South Sudan 2, Niger 1, Nigeria 5, Sultan 1, Tanzania 1, Uganda 1, Chad 1 | 36.56±10.97  24-56  16/0  Chinese  China 16 |

^*^ Median ± SD.

Patients suffered from falciparum malaria (FM); healthy individuals (Health);
