# Supplementary material for: Down-Regulation of Tim-3 in Monocytes and Macrophages in Plasmodium Infection and Its Association with Parasite Clearance
Source: Front Microbiol. 2017 Aug 2;8:1431. doi: 10.3389/fmicb.2017.01431 (PMC5539084; doi:10.3389/fmicb.2017.01431)
Supplement: Supplementary Table 2 — Primers for murine genes. [file Table2.DOC]

Supplemental Table 2. Primers for murine genes

| Gene | Forward | Reverse |
| --- | --- | --- |
| CD36  ICAM-1  VCAM-1  PECAM-1  TNF-α  iNOS  IL-12  Arg1  IL-10  β-actin  GAPDH | CTTTGGCTTAATGAGACTGGGAC  GTGATGCTCAGGTATCCATCCA  GTTCCAGCGAGGGTCTACC  ACGCTGGTGCTCTATGCAAG  CAGGCGGTGCCTATGTCTC  TCCAGAAGCAGAATGTGACC  CAATCACGCTACCTCCTCTTTT  TGAAAGGAAAGTTCCCAGATG  GCTCTTACTGACTGGCATGAG  TGCGTGACATCAAAGAGAAG  AGGTCGGTGTGAACGGATTTG | GCAACAAACATCACCACACCA  CACAGTTCTCAAAGCACAGCG  AACTCTTGGCAAACATTAGGTGT  TCAGTTGCTGCCCATTCATCA  CGATCACCCCGAAGTTCAGTAG  GGACCAGCCAAATCCAGTC  CAGCAGTGCAGGAATAATGTTTC  GTTCCCCAGGGTCTACGTCT  CGCAGCTCTAGGAGCATGTG  TCCATACCCAAGAAGGAAGG  TGTAGACCATGTAGTTGAGGTCA |

CD36, cluster of differentiation 36; ICAM-1, intercellular adhesion molecule; VCAM-1, vascular cell adhesion molecule; PECAM-1, platelet endothelial cell adhesion molecule; TNF-α, tumor necrosis factor-α; iNOS, inducible Nitric Oxide Synthase; IL-12, interleukine-12; Arg1, arginase-1; IL-10, interleukin-10; GAPDH, glyceraldehyde-3-phosphate dehydrogenase.
